# The impact of comorbidity on mortality in multiple myeloma: a Danish nationwide population‐based study

**DOI:** 10.1002/cam4.1128

**Published:** 2017-06-22

**Authors:** Henrik Gregersen, Annette Juul Vangsted, Niels Abildgaard, Niels Frost Andersen, Robert Schou Pedersen, Ulf Christian Frølund, Carsten Helleberg, Bettina Broch, Per Trøllund Pedersen, Peter Gimsing, Tobias Wirenfeldt Klausen

**Affiliations:** ^1^ Department of Hematology Aalborg University Hospital DK‐9000 Aalborg Denmark; ^2^ Department of Hematology Rigshospitalet University of Copenhagen DK‐2100 Copenhagen Denmark; ^3^ Department of Hematology and the Academy of Geriatric Cancer Research (AgeCare) Odense University Hospital DK‐5000 Odense C Denmark; ^4^ Department of Hematology Aarhus University Hospital DK‐8000 Aarhus Denmark; ^5^ Department of Hematology Regional Hospital West Jutland DK‐7500 Holstebro Denmark; ^6^ Department of Hematology Roskilde Hospital DK‐4000 Roskilde Denmark; ^7^ Department of Hematology Herlev Hospital DK‐2730 Herlev Denmark; ^8^ Department of Hematology Vejle Hospital DK‐7100 Vejle Denmark; ^9^ Department of Hematology Esbjerg Hospital DK‐6700 Esbjerg Denmark

**Keywords:** Comorbidity, international classification of diseases, multiple myeloma, prognosis, survival

## Abstract

To describe the prevalence of comorbidity and its impact on survival in newly diagnosed multiple myeloma patients compared with population controls. Cases of newly diagnosed symptomatic multiple myeloma during the 2005–2012 period were identified in the Danish National Multiple Myeloma Registry. For each myeloma patient, 10 members of the general population matched by age and sex were chosen from the national Civil Registration System. Data on comorbidity in the myeloma patients and the general population comparison cohort were collected by linkage to the Danish National Patient Registry (DNPR). Cox proportional hazards regression models were used to evaluate the prognostic significance of comorbidity. The study included 2190 cases of multiple myeloma and 21,900 population controls. The comorbidity was increased in multiple myeloma patients compared with population controls, odds ratio (OR) 1.4 (1.1–1.7). The registration of comorbidity was highly increased within the year preceding diagnosis of multiple myeloma (OR 3.0 [2.5–3.5]), which was attributable to an increased registration of various diseases, in particular, renal disease with OR 11.0 (8.1–14.9). The median follow‐up time from diagnosis of multiple myeloma for patients alive was 4.3 years (interquartile range 2.4–6.3). Patients with registered comorbidity had increased mortality compared with patients without comorbidity, hazard ratio 1.6 (1.5–1.8). Multiple myeloma patients have increased comorbidity compared with the background population, in particular during the year preceding the diagnosis of myeloma.

## Introduction

The survival of multiple myeloma patients has improved over the last two decades with the introduction of new drugs 1, 2. In addition, the concept of combined therapies has evolved in pursuit of synergistic effects 3. Although new treatment options have led to improved survival, multiple myeloma remains an incurable malignant disease and an important challenge is to apply data from clinical trials to the real‐life population of multiple myeloma 4.

The incidence of multiple myeloma increases with age and the prolonged survival of the population in general will lead to an increased number of multiple myeloma patients in the future 5. Comorbidities correlate with aging and make the elderly particularly vulnerable to toxicities of therapy. A key issue is therefore to choose the optimal therapy for these patients 6. An increasing awareness of the vulnerability of the elderly has lead to recommendations for dose adjustment of treatment in patients with comorbid conditions 7. Recently, a frailty score was proposed by the International Myeloma Working Group including age, geriatric assessment, and comorbidity assessed by the Charlson Comorbidity Index 8. This scoring system was based on data from three prospective clinical trials and predicts mortality and the risk of toxicity in myeloma patients. However, patients with comorbidities are often excluded from clinical trials and therefore these data may not necessarily provide guidance for treatment decisions for the elderly patients in general. Data on the prevalence and impact of comorbidity in the real‐life population of multiple myeloma patients is limited 5, 9.

We therefore conducted a population‐based Danish study of all patients with newly diagnosed multiple myeloma and a matched control population to compare the burden of comorbidity and to evaluate the impact of comorbidity on mortality.

## Materials and Methods

Denmark has approximately 5.6 million inhabitants and every resident is registered in the national Civil Registration System (CRS), and is assigned a personal identification number (the CPR‐number) at birth, which allows linkage between demographic and medical registries 10. The National Health Service provides tax‐supported health care for all citizens in Denmark and guarantees free access to hospitals.

### Study population

The Danish Multiple Myeloma Registry was established in 2005 by the Danish Myeloma Study Group (DMSG) 11. Danish hematology departments are obligated to report all incident cases of multiple myeloma, solitary plasmacytoma, MGUS, and plasma cell leukemia to the registry. The database include the date of myeloma diagnosis, percentage of plasma cells in the bone marrow, result of skeletal X‐ray, ECOG performance status, clinical presentation, for example, spinal cord compression, type and concentration of the M‐component, and basic biochemical parameters, for example, beta‐2‐microglobulin. In the study period, the 2003 criteria for classification of monoclonal gammopathies from the International Myeloma Working Group were used 12. Annual links to the Danish National Patient Registry ensure that cases of multiple myeloma that have not been reported to the Danish Multiple Myeloma Registry are subsequently included. A recent validation of the registry has shown almost 100% completeness 11.

### Data on comorbidity

The Danish National Patient Registry (DNPR) has, since 1977, routinely collected nationwide data on all nonpsychiatric hospitalizations, and since 1995, visits to emergency departments and outpatient clinics have also been included 13. The DNRP records CPR‐number and date of each hospital visit, together with primary and secondary discharge diagnoses. The DNPR has almost complete coverage of national hospital admissions 14. Data have been coded according to the *International Classification of Diseases*, 10th revision (ICD‐10) since 1994.

Comorbidity was classified according to the 19 diseases included in the Charlson Comorbidity Index (CCI) 15. We computed a CCI score based on registered diagnoses within 10 years and until 1 month before diagnosis of multiple myeloma. Four levels of comorbidity were defined: 0 (“low”) for individuals with no recorded underlying diseases included in the CCI; 1 (“moderate low”), 2 (“moderate high”), and ≥3 (“high”).

### General population comparison cohort

For each myeloma patient, we randomly chose 10 members of the general population from the CRS, matched by year of birth and sex. The members of the comparison cohort had to be alive with no diagnosis of multiple myeloma on the date when follow‐up of the matched myeloma patient began. Thus, the control persons were included at the time of diagnosis of their corresponding multiple myeloma patient with whom they were matched. Data on comorbidity was obtained from the DNPR.

### Statistical analysis

Comparisons of binary variables were conducted by chi‐square test or Fisher's exact test, mid‐P approach 16. Continuous variables were presented using medians with range or interquartile range. Ordinal variables were compared using Mann–Whitney test. Overall survival was defined as the time from diagnosis to death from any cause and was described using the Kaplan–Meier method. Comorbidity was assessed for two different time periods, namely from 1 year until 1 month before diagnosis of multiple myeloma and from 10 years until 1 month before diagnosis of multiple myeloma. Cox proportional hazards regression models were performed to evaluate the prognostic significance of comorbidity and the results are presented as estimated hazard ratios (HRs) with 95% confidence intervals. The proportionality assumptions for the models were assessed by scaled Schoenfeld residuals and we did not find violations of the assumptions. When comparing subgroups with few events, a permutation method was used for calculating *P*‐values 17. R function “permlogrank” in the “clinfun” package was used for these calculations. The interaction between myeloma and comorbidities on mortality was inspected by calculating an interaction contrast on an additive scale. The age/gender standardized mortality rate differences between multiple myeloma and population controls for each level of comorbidity were calculated and compared to the difference in multiple myeloma/population controls without comorbidities 18. For calculations of nonlinear effects on survival of age, a general additive model was performed with age as a smoothing spline with four degrees of freedom. All tests were two‐sided and *P*‐values of 0.05 were considered statistically significant. All confidence intervals are 95% and two‐sided. Data analyses were performed using R version 3.2.3 (R Foundation for Statistical Computing, Vienna, Austria).

The study was approved by the Danish Data Protection Agency (No. 2008‐58‐0028).

## Results

The study included a total of 2190 patients with symptomatic multiple myeloma in the period 2005–2012 in Denmark. The median age at diagnosis was 70 years (range 30–98 years), and 1204 (55%) were men. The median duration of follow‐up for patients alive was 4.3 years (interquartile range 2.4–6.3). Characteristics of the patients and population controls are described in Table 1.

**Table 1:** Characteristics of 2190 Danish patients with newly diagnosed symptomatic multiple myeloma and 21,900 matched population controls

		Myeloma patients	Population controls
Total		2190	21,900
Gender, *n* (%)	Male	1204 (55%)	12,040 (55%)
Age (years)	Median (min, max)	70 (19 – 98)	70 (19–98)
Age >65 years, *n* (%)		1425 (65%)	14,250 (65%)
M‐component, *n* (%)	IgA	443 (20.8%)	–
	IgG	1184 (55.5%)	–
	IgM	11 (0.5%)	–
	Free light chain	339 (15.9%)	–
	Other^a^	156 (7.4%)	–
	Missing	57	
International staging system, *n* (%)	I	497 (27.4%)	–
	II	651 (36.7%)	–
	III	639 (34.9%)	–
	Missing	403	
WHO performance status, *n* (%)	0	502 (23.0%)	–
	1	888 (40.8%)	
	2	442 (20.3%)	–
	≥3	347 (15.9%)	–
	Missing	11	
LDH, increased, *n* (%)		463 (22.2%)	
Missing		104	
CRP, increased, *n* (%)		809 (38.6%)	
Missing		92	
Creatinine, increased, *n* (%)		770 (35.6%)	
Missing		27	
Comorbidity score^b^	0	1294 (59.1%)	14250 (65.1%)
	1	317 (14.5%)	3278 (15.0%)
	2	306 (14.0%)	2403 (11.0%)
	≥3	273 (12.5%)	1969 (9.0%)

a Biclonal and nonsecretory myeloma.

b Comorbidity classified according to Charlson Comorbidity Index (*P* < 0.0001).

### Comorbidity in the myeloma cohort and population controls

Comorbidity at cohort entry was registered in 40.9% of the patients. The overall comorbidity was increased in multiple myeloma patients compared with population controls, odds ratio (OR) 1.4 (1.1–1.7). The prevalence of comorbidity was higher in patients over 65 years of age compared with the younger patients, for example, three or more comorbidities in 226 (15.9%) patients >65 years versus 47 (6.1%) patients ≤65 years (*P* < 0.0001). In addition, comorbidity was higher in male patients (42.3%) compared with female patients (39.3%) (*P* = 0.04). Table 2 describes details on registration of the diseases in the Charlson Comorbidity Index. The registration of comorbidity was highly increased within the year preceding diagnosis of multiple myeloma (OR 3.0 [2.5–3.5; *P* < 0.0001]) which was attributable to an increased registration of various diseases, in particular, renal disease with OR 11.0 (8.1–14.9) (Table 2). By contrast, comorbidity was not increased in the time period from 10 years until 1 year before diagnosis of multiple myeloma (OR 1.0 [0.9–1.1, *P* = 0.77]) and the only deviations of individual diseases in this time period were an increased prevalence of renal disease (OR 1.5 [1.1–2.1; *P* = 0.02]) and lower prevalence of dementia (OR 0.3 [0.2–0.6; *P* = 0.0001]). An increased registration of venous thromboembolism was observed in the period from 10 years until diagnosis of multiple myeloma and in the year preceding the diagnosis of multiple myeloma with OR of 1.5 (1.1–1.9; *P* = 0.003) and 2.8 (1.6–4.5; *P *= 0.0004), respectively.

**Table 2:** Hazard ratios comparing the individual diseases included in the Charlson Comorbidity Index in 2190 Danish patients with newly diagnosed symptomatic multiple myeloma and 21,900 population controls

Diagnosis	Registered comorbidity from 10 years until 1 month before diagnosis of multiple myeloma			Registered comorbidity from 1 year until 1 month before diagnosis of multiple myeloma		
	Number (%)	OR (95% CI)	*P*	Number (%)	OR (95% CI)	*P*
Any Charlson condition	896 (40.9%)	1.4 (1.1–1.7)	<0.0001	194 (8.9%)	3.0 (2.5–3.5)	<0.0001
Myocardial infarction	118 (5.4%)	1.1 (0.9–1.3)	0.5745	18 (0.8%)	1.7 (1.0–2.7)	0.0557
Congestive heart failure	126 (5.8%)	1.4 (1.1–1.7)	0.0016	36 (1.6%)	2.8 (1.9–4.0)	<0.0001
Peripheral vascular disease	82 (3.7%)	0.9 (0.7–1.1)	0.3126	16 (0.7%)	1.2 (0.7–1.9)	0.5429
Cerebrovascular disease	160 (7.3%)	0.9 (0.7–1.0)	0.1390	29 (1.3%)	1.3 (0.9–1.9)	0.2181
Dementia	17 (0.8%)	0.5 (0.3–0.8)	0.0015	8 (0.4%)	1.1 (0.5–2.1)	0.8539
Chronic pulmonary disease	147 (6.7%)	1.0 (0.8–1.2)	0.9526	28 (1.3%)	1.7 (1.1–2.5)	0.0140
Connective tissue disease	72 (3.3%)	1.2 (0.9–1.5)	0.2264	19 (0.9%)	3.9 (2.2–6.6)	<0.0001
Ulcer disease	89 (4.1%)	1.6 (1.3–2.0)	0.0002	33 (1.5%)	5.3 (3.4–8.1)	<0.0001
Mild liver disease	17 (0.8%)	1.2 (0.7–2.0)	0.4267	6 (0.3%)	3.2 (1.2–7.7)	0.0276
Diabetes Mellitus	71 (3.2%)	1.0 (0.8–1.3)	0.7061	12 (0.5%)	1.6 (0.8–2.8)	0.1664
Hemiplegia	7 (0.3%)	1.3 (0.6–2.8)	0.4779	1 (0.0%)	2.2 (0.1–14.5)	0.5324
Moderate and severe renal disease	131 (6.0%)	3.7 (3.0–4.6)	<0.0001	89 (4.1%)	11.0 (8.1–14.9)	<0.0001
Diabetes mellitus with chronic complications	73 (3.3%)	1.1 (0.9–1.4)	0.3096	18 (0.8%)	2.3 (1.3–3.7)	0.0042
Any tumor	223 (10.2%)	1.2 (1.0–1.4)	0.0149	49 (2.2%)	1.9 (1.3–2.5)	0.0003
Leukemia	8 (0.4%)	1.1 (0.5–2.1)	0.8539	2 (0.1%)	1.6 (0.2–6.0)	0.5517
Lymphoma	21 (1.0%)	2.0 (1.2–3.2)	0.0069	8 (0.4%)	5.8 (2.3–13.6)	0.001
Moderate and severe Liver disease	2 (0.1%)	0.5 (0.1–1.7)	0.3254	1 (0.0%)	1.9 (0.1–11.3)	0.6145
Metastatic solid tumor	41 (1.9%)	1.9 (1.3–2.6)	0.0006	25 (1.1%)	4.1 (2.6–6.5)	<0.0001
AIDS	0 (0.0%)	–	0.62	0 (0.0%)	–	–

### Survival in the multiple myeloma cohort

**Figure 1:**
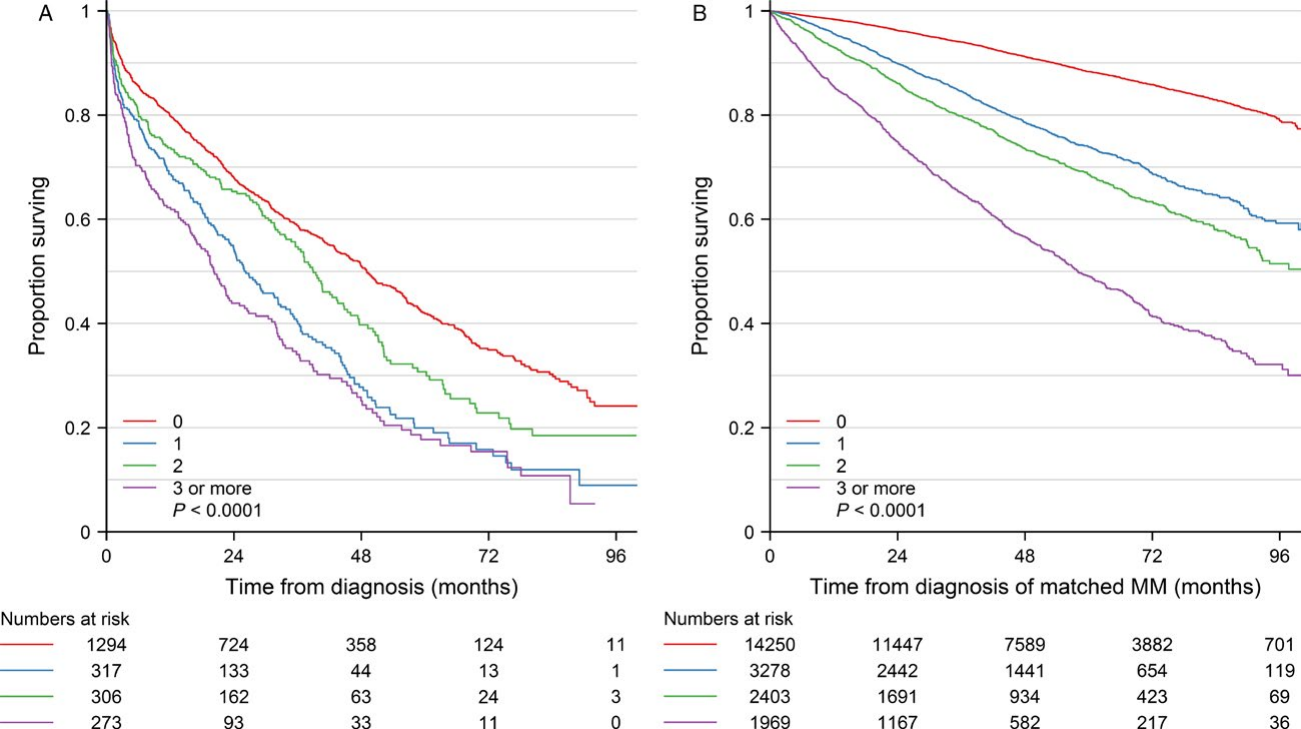
Survival according to Charlson comorbidity score for 2190 patients with newly diagnosed symptomatic multiple myeloma (MM) and 21,900 population controls in Denmark during the period 2005–2012.

The median overall survival in all patients was 3.3 years (1.0–6.5) comprising median survival of 5.7 years (2.5 – not reached) and 2.3 years (0.6–4.7) in patients aged 65 years or younger and in patients older than 65 years, respectively. Figure 1 shows the survival stratified according to the Charlson Comorbidity Index for myeloma patients and population controls. Patients with any registered comorbidity had increased mortality compared with patients without comorbidity, HR 1.6 (1.5–1.8). The mortality according to the diseases that constitute the Charlson Comorbidity Index is shown in Table 3. Registration of venous thromboembolism was associated with increased mortality (HR 2.0 [1.5–2.7; *P* < 0.0001]).

**Table 3:** Mortality according to comorbidities used in the Charlson Comorbidity Index in 2190 Danish newly diagnosed symptomatic multiple myeloma patients

Diagnosis	5‐year survival	Hazard ratio	95% CI	*P*
Any Charlson condition	23%	1.6	1.5–1.8	<0.0001
Myocardial infarction	22%	1.6	1.3–2.1	<0.0001
Congestive heart failure	20%	1.8	1.5–2.2	<0.0001
Peripheral vascular disease	12%	1.6	1.2–2.1	0.0005
Cerebrovascular disease	20%	1.6	1.3–1.9	<0.0001
Dementia	0%	2.8	1.7–4.8	0.0004
Chronic pulmonary disease	16%	1.7	1.4–2.1	<0.0001
Connective tissue disease	30%	1.2	0.9–1.6	0.16
Ulcer disease	13%	1.8	1.4–2.3	<0.0001
Mild liver disease	0%	1.8	1.0–3.1	0.04
Diabetes mellitus	17%	1.5	1.1–2.0	0.010
Hemiplegia^a^	19%	–	–	0.90
Moderate and severe renal disease	24%	1.6	1.2–1.9	<0.0001
Diabetes mellitus with chronic complications	18%	1.5	1.2–2.1	0.003
Any tumor	26%	1.2	1.0–1.4	0.06
Leukemia^a^	44%	–	–	0.90
Lymphoma	25%	1.2	0.7–2.0	0.55
Moderate and severe Liver disease^a^	50%	–	–	0.71
Metastatic solid tumor	22%	1.2	0.8–1.7	0.35

The Hazard ratios were based on comparisons with patients without the particular comorbidity. The analysis included registered comorbidity from 10 years until 1 month before diagnosis of multiple myeloma. Survival was defined as the time from diagnosis of multiple myeloma to death from any cause.

a For uncommon comorbidities (*n *< 10), no Hazard ratios were calculated and *P*‐values were calculated using Monte Carlo method. No multiple myeloma patients had AIDS and this condition was excluded from the table.

### Risk factors for survival

A Cox proportional hazards model was used to analyze the impact on mortality of comorbidity and well‐known risk factors, including age, International Staging System, serum lactate dehydrogenase, serum creatinine, serum C‐reactive protein, IgA M‐component, and WHO performance status (0–1 vs. 2–4) (Table 4). The mortality was increased in patients registered with comorbidity according to Charlson Comorbidity Index compared with patients without registered comorbidity, for example, hazard ratios of 1 (ref), 1.5 (1.2–1.8), 1.3 (1.0–1.5), and 1.7 (1.4–2.1) in the comorbidity groups 0, 1, 2, and ≥3, respectively. A division of comorbidity into two groups similar to the International Myeloma Working Group (IMWG) frailty study (CCI <2 vs. CCI ≥2) showed an increased mortality in the group with high comorbidity (HR 1.3 [1.1–1.5; *P* = 0.0003]) 8. Three sensitivity analyses on the association between OS and comorbidities were performed. The analyses were (1) using CCI excluding renal disease, (2) using CCI excluding comorbidities with onset less than a year before multiple myeloma diagnosis, and (3) including age as a continuous nonlinear covariate. All models showed comparable effect of comorbidities on overall survival (data not shown).

**Table 4:** Multivariate analysis of prognostic factors on survival in 2190 Danish patients with newly diagnosed symptomatic multiple myeloma

Variable	Univariate analysis	Multivariable analysis
	Hazard ratio (95% CI)	Hazard ratio (95% CI)
Charlson Comorbidity Index		
0	1 (ref)	1 (ref)
1	1.7 (1.5–2.0)	1.5 (1.2–1.8)
2	1.3 (1.1–1.5)	1.3 (1.0–1.5)
≥3	2.0 (1.7–2.4)	1.7 (1.4–2.1)
Age (>65 years)	2.5 (2.2–2.9)	2.2 (1.9–2.6)
Gender (male)	1.0 (0.9–1.1)	–
International staging system (ISS)		
1	1 (ref)	1 (ref)
2	1.8 (1.5–2.2)	1.6 (1.3–1.9)
3	2.9 (2.4–3.4)	1.9 (1.6–2.4)
Increased serum lactate dehydrogenase level	1.5 (1.3–1.7)	1.3 (1.1–1.5)
Increased serum creatinine level	1.8 (1.6–2.0)	1.2 (1.0–1.4)
Increased serum C‐reactive protein level	1.6 (1.5–1.8)	1.2 (1.1–1.4)
IgA M‐component	1.2 (1.0–1.4)	1.3 (1.1–1.5)
WHO Performance status (0–1 vs. 2–4)	2.0 (1.8–2.2)	1.7 (1.5–2.0)

Comorbidity assessed by Charlson Comorbidity Index based on diagnoses registered from 10 years to 1 month prior to myeloma diagnosis in the Danish National Patient Registry.

### Comorbidity and WHO performance status

There was no association between Charlson Comorbidity Index and the WHO performance status at time of diagnosis of multiple myeloma (*p* = 0.07, chi‐square test) (Table 5).

**Table 5:** Association between comorbidity and WHO performance status in 2190 Danish patients with newly diagnosed symptomatic multiple myeloma

Charlson comorbidity Index	WHO performance status 0–1	WHO performance status 2–4
0	838 (65.0%)	451 (35.0%)
1	191 (60.6%)	124 (39.4%)
2	204 (66.9%)	101 (33.1%)
3	157 (58.1%)	113 (41.9%)

*P *= 0.07 (chi‐square test). Numbers do not add up to 2190 due to missing data on WHO performance status.

### High‐dose melphalan with hematopoietic stem cell support

High‐dose melphalan with haematopoietic stem cell support (HDT) was used as first‐line therapy in 582 (76.1%) patients 65 years or younger, and in 103 (7.2%) patients older than 65 years. Comorbidity affected the utilization of HDT, for example, used in 452 (81.1%) patients 65 years or younger with no registered comorbidity in contrast to 24 (51.1%) patients with three or more comorbidities (Table 6).

**Table 6:** Use of treatment with high‐dose melphalan with stem cell support (HDT) according to comorbidity in 2190 Danish patients with newly diagnosed symptomatic multiple myeloma

Comorbidity	All patients		Patients age ≤65 years	
	HDT	Other treatment	HDT	Other treatment
0	521 (76.1%)	773 (51.4%)	452 (77.7%)	105 (57.4%)
1	64 (9.3%)	253 (16.8%)	49 (8.4%)	28 (15.3%)
2	73 (10.7%)	233 (15.9%)	58 (10.0%)	26 (14.2%)
≥3	27 (3.9%)	246 (16.3%)	23 (4.0%)	24 (13.1)
*P* a		<0.0001		<0.0001

Comorbidity according to Charlson Comorbidity index. Chi‐square test. HDT: intention‐to‐treat.

a HDT versus no HDT for all patients and for patients with age ≤65 years.

## Discussion

In this large nationwide cohort study, we found an increased comorbidity in patients with multiple myeloma at time of diagnosis compared to population controls. This increase in comorbidity was mainly confined to the year proceeding diagnosis of myeloma. In both younger and elderly myeloma patients, comorbidity was associated with increased mortality.

The prevalence of comorbid diseases in our study is in accordance with the observations in a Swedish population‐based study based on the Swedish Cancer Registry where comorbidity was seen in approximately 40% of the multiple myeloma patients and with an Italian study based on a regional multiple myeloma registry 9, 19. We provide a further extension of the importance of comorbidity by comparing comorbidity in multiple myeloma patients with population controls and we found an increased registration of various comorbidities in multiple myeloma patients, in particular renal disease. This finding is consistent with studies on solid cancers that observed an increased comorbidity compared to the background population at diagnosis in specific cancers, for example, in lung and colorectal cancer patients 8, 20. In our study, the increased comorbidity was mainly caused by an increased registration of comorbid diseases within the last year preceding the diagnosis of multiple myeloma. Several factors may be important and involved in this observation. Well‐known multiple myeloma complications and morbidity secondary to multiple myeloma may partly explain the increased frequency, for example, the increased risk of myocardial infarction within the first years might reflect the increased risk of thromboembolism observed in multiple myeloma 21. This is supported by the fact that we found the same pattern for venous thromboembolism in our study cohort. The high prevalence of renal disease compared to the population controls is plausible and reflects that renal impairment is a frequent complication in multiple myeloma 22. In addition, our cohort will have included cases of monoclonal gammopathy of renal significance (MGRS) that later underwent malignant transformation to multiple myeloma 23, 24. An essential issue is the distinction between multiple myeloma complications and true comorbidity. A considerable part of the registered comorbidity within the last year prior to diagnosis might be related to the diagnostic process rather than reflecting true comorbidity in multiple myeloma. In a few cases, it might reflect the detection of multiple myeloma in patients in diagnostic workup for unrelated diseases which are subsequently registered as part of the comorbidity. However, symptomatic multiple myeloma is not likely to pass undetected for longer periods of time due to the prominent symptomatology. The association between registration of comorbidity and time to diagnosis of multiple myeloma raises methodological issues regarding when to assess comorbidity in studies on multiple myeloma.

We found that comorbidity increased the mortality in multiple myeloma patients. There was no clear difference in mortality between the subgroups 1, 2, and ≥3 according to Charlson Comorbidity Index. However, differences in mortality were observed when comorbidity according to Charlson Comorbidity Index was used as dichotomous variables, for example, between patients with no registered comorbidity and patients with any grade of comorbidity, or between patients with comorbidity score less than 2 compared with patients with a score of 2 or higher. The HR of 1.3 in this later division is in accordance with the HR of 1.37 found in the IMWG frailty study by Palumbo et al. 8. This frailty score is based on data from clinical trials and due to study exclusion criteria, the pattern of comorbidity is likely to be different from that of unselected myeloma patients in a population‐based study. Thus, our study provides support for the comorbidity element of the IMWG frailty score also being valid in a population‐based setting. However, there is a need for studies that validate the frailty score and address comorbidity in population‐based settings.

Charlson Comorbidity Index creates a pooled estimate of comorbidity and assigns particular weights to a number of diseases 15. It was developed almost 30 years ago to predict mortality in medical patients admitted to hospitals and has been shown to be robust in a number of clinical settings, including validated for several different cancers and it is the most extensively studied comorbidity index for predicting mortality. However, the prognosis of the individual included diseases has changed over time, for example, the prognosis of AIDS has considerably changed since the eighties and some of the comorbidities are rarely detected in myeloma patient, for example, dementia and AIDS. In addition, the Charlson Comorbidity Index only includes limited information on disease severity. A recent study indicates that the Freiburg comorbidity index which is based on performance status, renal impairment, and lung disease might be more useful than the Charlson Comorbidity Index in predicting overall survival in elderly patients with multiple myeloma 25.

The adverse prognostic impact of comorbidity on survival is complex. Comorbidities may independently increase the risk of death or might add to well‐known myeloma risk factors, for example, further increase the risk of serious infections or venous thromboembolic disease. In addition, comorbidity may indirectly affect the prognosis by affecting the choice of myeloma treatment and lead to more frequent dose reduction 26. Our data confirm how a high burden of comorbidity reduces the use of HDT in younger myeloma patients. Our study design did not permit an evaluation of the association between comorbidity and the use of other myeloma treatment and dose reductions.

The main strength of our study is its large size with inclusion of all newly diagnosed multiple myeloma patients in a population‐based setting and the use of a population comparison cohort. The risk of selection bias in this setting is negligible which contrasts studies including patients participating in clinical trials or patient series from single centers. We included the very old and frail patients and our study reflects the real‐life population of multiple myeloma patients in Denmark. Consequently, the mortality of the elderly patients is higher than reported in most other studies 27, 28, 29. Furthermore, the use of DNPR data ensures a complete nationwide ascertainment of former comorbid conditions in the myeloma patients and the comparison cohort. Despite these advantages, our registry‐based study has some limitations. We had data on ISS stage in most patients, whereas cytogenetic and FISH only were performed in a limited number of the patients, in particular, in the early part of the inclusion period. Consequently, we were not able to include this important prognostic factor in our analysis of factors affecting mortality. The DNPR provides limited data on the severity and duration of the comorbid conditions and we were not able to validate the proposed Freiburg index. However, the positive predictive values in the DNPR of the diagnosis groups included in the Charlson Comorbidity Index are high (overall 98.0%) 30. Diseases and complications diagnosed in primary health care are not registered in the DNPR and this under‐registration could diminish potential effects of specific conditions. Another concern is the risk of coding errors leading to misclassification of comorbidity. However, this bias is likely to be similar in the myeloma patients and populations controls at the time of interest and not subject to the surveillance bias that might follow the diagnosis of multiple myeloma.

In conclusion, we found increased registration of comorbid disease within the year prior to diagnosis of multiple myeloma. This finding is likely to represent complications and morbidity secondary to yet undiagnosed multiple myeloma and also conditions related to the diagnostic process but it underlines the potential burden of comorbidity in myeloma. We found that comorbidity increased the mortality in multiple myeloma patients when we assessed the comorbidity in the Charlson Comorbidity Index as dichotomous variables, for example, no comorbidity versus any comorbidity or comorbidity score less than 2 compared with a score of 2 or higher which supported the use of the index in the IMWG frailty study. However, we did not find any clear difference in mortality between the subgroups 1, 2, and ≥3 according to Charlson Comorbidity Index.

## Conflict of Interest

None declared.
